# *TP53* and *TP53*-associated genes are correlated with the prognosis of paediatric neuroblastoma

**DOI:** 10.1186/s12863-022-01059-5

**Published:** 2022-06-02

**Authors:** Haiwei Wang, Xinrui Wang, Liangpu Xu, Ji Zhang

**Affiliations:** 1 Medical Research Center, Fujian Maternity and Child Health Hospital, Fuzhou, Fujian China; 2 State Key Laboratory for Medical Genomics, Shanghai Institute of Hematology, Rui-Jin Hospital Affiliated to School of Medicine, Shanghai Jiao Tong University, Shanghai, China

**Keywords:** Paediatric neuroblastoma, *TP53*, *CCNE1*, *CDK2*, *CHEK2*, *SESN1*

## Abstract

**Background:**

*TP53* is rarely mutated in paediatric neuroblastoma. The prognosis of *TP53* and *TP53*-associated genes in paediatric neuroblastoma is unclear. The objectives of the study were to analyse datasets of 2477 paediatric neuroblastoma patients from eight independent cohorts to reveal the prognosis of *TP53* and *TP53*-associated genes.

**Results:**

High *TP53* mRNA expression was associated with shortened event-free survival and overall survival in paediatric neuroblastoma. Moreover, a higher enrichment score of the *TP53* signalling pathway was associated with worse clinical outcomes of paediatric neuroblastoma. Among the genes associated with *TP53*, *CCNE1*, *CDK2* and *CHEK2* were correlated with unfavourable clinical outcomes, while *SESN1* was correlated with favourable clinical outcomes of paediatric neuroblastoma in the eight independent neuroblastoma cohorts. *TP53*, *CCNE1*, *CDK2* and *CHEK2* were overexpressed in neuroblastoma patients with *MYCN* amplification, while *SESN1* was downregulated in neuroblastoma patients with *MYCN* amplification. *CCNE1*, *SESN1*, *MYCN* amplification and age at diagnosis were independent prognostic markers of neuroblastoma. *CCNE1* was also highly expressed in paediatric neuroblastoma patients with an age at diagnosis ≥ 18 months, while *SESN1* was downregulated in paediatric neuroblastoma patients with an age at diagnosis ≥ 18 months. Combinations of *CCNE1* with age at diagnosis or combinations of *SESN1* with age at diagnosis achieved superior prognostic effects in paediatric neuroblastoma. Finally, we constructed a nomogram risk model of paediatric neuroblastoma based on age and *TP53*, *CCNE1*, *CDK2*, *CHEK2* and *SESN1* expression. The nomogram model could predict the overall survival of paediatric neuroblastoma and *MYCN* nonamplified paediatric neuroblastoma with high specificity and sensitivity.

**Conclusions:**

*TP53* and *TP53*-associated genes *CCNE1*, *CDK2*, *CHEK2* and *SESN1* were significantly associated with the clinical outcomes of paediatric neuroblastoma.

**Supplementary Information:**

The online version contains supplementary material available at 10.1186/s12863-022-01059-5.

## Introduction

Paediatric neuroblastoma is the most common extracranial malignant tumour type in children [1, 2]. The aggressiveness and clinical outcomes of children with neuroblastoma are significantly different [3]. In 2009, the International Neuroblastoma Risk Group identified 13 prognostic factors of neuroblastoma through a large-scale cohort study, including age at diagnosis, tumour stage, histological type, degree of differentiation and *MYCN* amplification [4]. *MYCN* genetic amplification occurs in 25% of neuroblastoma [5] and is an unfavourable prognostic factor of neuroblastoma [6]. Paediatric neuroblastoma patients with an age at diagnosis ≥ 18 months have a poor prognosis [7]. The high clinical stage has adverse effects on the prognosis of children with neuroblastoma [8]. These risk stratifications provide a reference basis for the prognosis and choice of treatment of neuroblastoma. However, additional molecular biomarkers are still needed to provide better classifications and prognoses of paediatric neuroblastoma.

*TP53* is referred to as the "guardian of the genome", and almost all key cellular activities are related to *TP53* functions, such as apoptosis, cell cycle regulation, DNA repair and cell metabolism [9]. As a critical tumour suppressor, *TP53* mutations have been identified in half of adult cancer patients [10]. Mutated *TP53* induces tumour angiogenesis and promotes tumour metastasis [11, 12]. *TP53* mutation is a poor prognostic factor for adult tumour patients [13]. Approximately 50% of relapsed adult tumours are correlated with *TP53* loss of function [14, 15]. Restoration of mutant *TP53* to a normal state represents a potential therapeutic concept for the treatment of adult tumours [16]. However, *TP53* is rarely mutated in paediatric neuroblastoma [17, 18], and the functions of *TP53* in paediatric neuroblastoma are largely unclear.

In addition to genetic mutations, *TP53* may be involved in paediatric neuroblastoma through other mechanisms. Some variants of *TP53* are associated with susceptibility to neuroblastoma [19]. *TP53* protein frequently accumulates in neuroblastoma cells [20]. Moreover, *TP53* is a target of *MYCN*. *MYCN* can directly bind to the *TP53* promoter regions and upregulate *TP53* mRNA expression [21]. Loss of *TP53* function induces radioresistance in neuroblastoma by regulating metabolism [22]. In addition, the *TP53* partner gene *MDM2* is also a *MYCN* transcriptional target and is implicated in neuroblastoma pathogenesis [23, 24]. *MDM2* overexpression maintains *MYCN* stabilization and translation in paediatric neuroblastoma cells [24] and represents an unfavourable prognostic factor of neuroblastoma [25]. Inhibition of *MDM2* suppresses the progression of *MYCN*-dependent neuroblastoma [26]. *MDM2*-*TP53* antagonists represent potential therapeutic drugs for paediatric neuroblastoma [27, 28]. Except for *MDM2*, *TP53* is correlated with multiple other genes [29, 30]. However, in paediatric neuroblastoma, the prognosis of *TP53* and its associated genes is still unclear.

Using the public paediatric neuroblastoma cohorts in Therapeutically Applicable Research to Generate Effective Treatments (TARGET), European Bioinformatics Institute (EMBL-EBI) and Gene Expression Omnibus (GEO) datasets, we analysed the prognosis of *TP53* and *TP53*-associated genes in neuroblastoma. Our results suggested that *TP53* and its associated genes *CCNE1*, *CDK2*, *CHEK2*, and *SESN1* were significantly associated with the clinical event-free survival and overall survival of paediatric neuroblastoma. Combinations of *CCNE1* expression and *SESN1* expression with age at diagnosis achieved a better prognosis of neuroblastoma. Finally, we showed that a nomogram risk model based on age, *TP53*, *CCNE1*, *CDK2*, *CHEK2* and *SESN1* expression could predict the overall survival of paediatric neuroblastoma with high specificity and sensitivity.

## Results

### Higher *TP53* mRNA expression is associated with worse clinical outcomes of paediatric neuroblastoma

We collected public paediatric neuroblastoma cohorts and designed a study process to determine the prognostic effects of *TP53* and *TP53*-associated genes in paediatric neuroblastoma (Supplementary Fig. 1). First, the prognostic value of *TP53* expression, the *TP53* pathway and *TP53*-associated genes was determined using the TARGET, EMBL-EBI and GEO datasets. The independent prognostic factors in paediatric neuroblastoma were also determined. Finally, we also constructed a nomogram model to predict the overall survival of paediatric neuroblastoma based on age and *TP53*-associated genes.

**Fig. 1:**
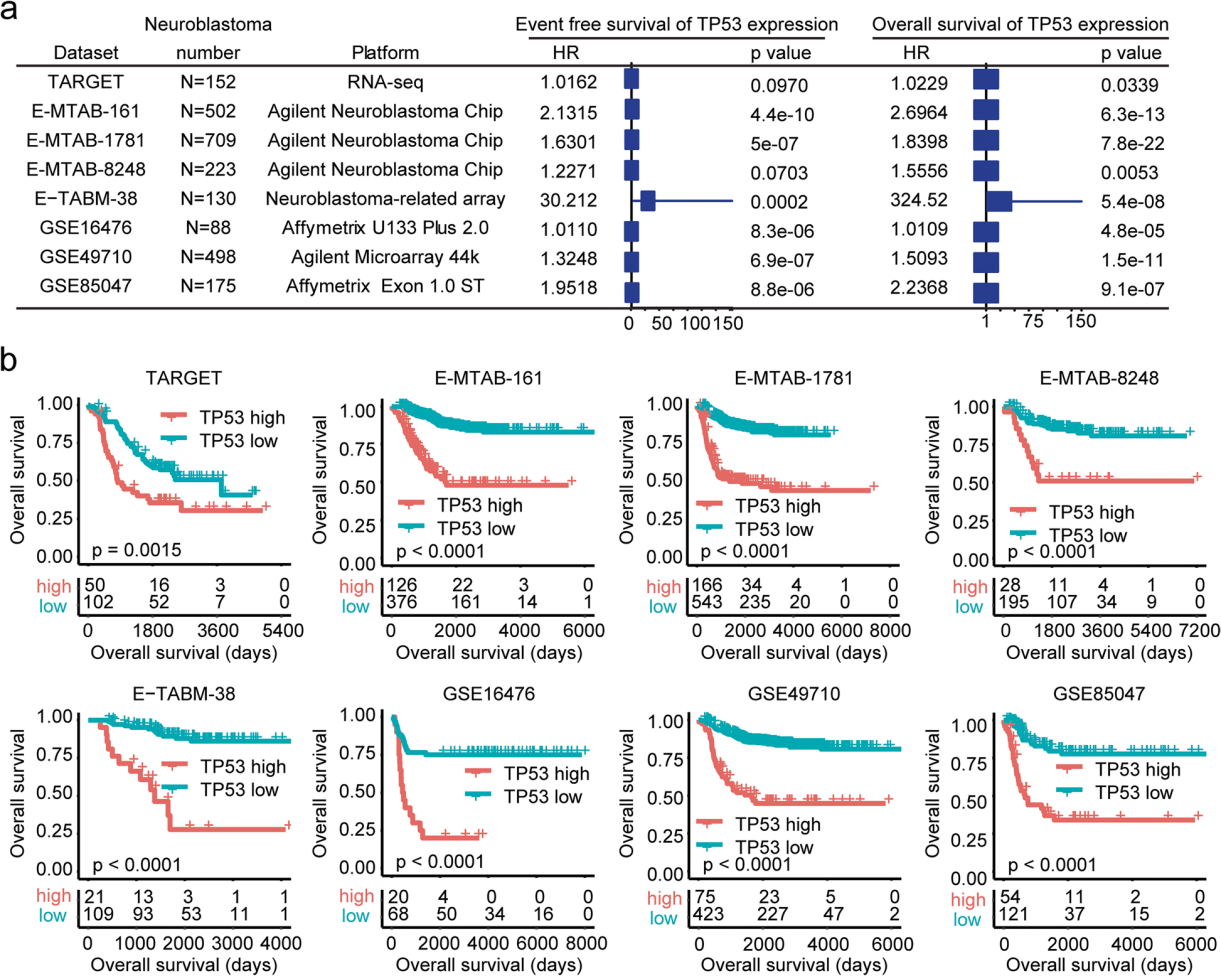
Higher *TP53* mRNA expression is associated with worse clinical outcomes of paediatric neuroblastoma. **a** Forest plots showing the associations of *TP53* mRNA expression with neuroblastoma event-free survival and overall survival in the TARGET, E-MTAB-161, E-MTAB-1781, E-MTAB-8248, E-TABM-38, GSE16476, GSE49710 and GSE85047 datasets. The number of paediatric neuroblastoma patients in each dataset and the gene microarray platform of each dataset are shown. **b** Different overall survival of paediatric neuroblastoma patients with higher or lower TP53 expression in the TARGET, E-MTAB-161, E-MTAB-1781, E-MTAB-8248, E-TABM-38, GSE16476, GSE49710 and GSE85047 datasets

In total, 152 paediatric neuroblastoma patients from the TARGET dataset along with 2325 paediatric neuroblastoma patients from the EMBL-EBI and GEO datasets, including the E-MTAB-161, E-MTAB-1781, E-MTAB-8248, E-TABM-38, GSE16476, GSE49710 and GSE85047 datasets, were collected (Fig. 1a). The number of paediatric neuroblastoma patients in each dataset and the gene microarray platform of each dataset are shown in Fig. 1a.

First, the prognostic effects of *TP53* mRNA expression in paediatric neuroblastoma were determined. Univariate Cox regression analysis suggested that *TP53* expression was associated with the event-free survival of paediatric neuroblastoma in the E-MTAB-161, E-MTAB-1781, E-TABM-38, GSE16476, GSE49710 and GSE85047 datasets (Fig. 1a). However, in the TARGET and E-MTAB-8248 datasets, *TP53* mRNA expression was not significantly associated with the event-free survival of paediatric neuroblastoma (Fig. 1a). Moreover, *TP53* expression was associated with the overall survival of paediatric neuroblastoma in all eight independent datasets: TARGET, E-MTAB-161, E-MTAB-1781, E-MTAB-8248, E-TABM-38, GSE16476, GSE49710 and GSE85047 (Fig. 1a).

Furthermore, paediatric neuroblastoma patients in each dataset were divided into *TP53* higher and lower subgroups based on the mRNA expression levels of *TP53*. The different clinical outcomes of *TP53* higher and lower subgroups were determined by Kaplan–Meier survival analysis. Lower *TP53* expression was associated with prolonged event-free survival and overall survival of paediatric neuroblastoma in the TARGET, E-MTAB-161, E-MTAB-1781, E-MTAB-8248, E-TABM-38, GSE16476, GSE49710 and GSE85047 datasets (Fig. 1b and Supplementary Fig. 2). These results suggested that *TP53* mRNA expression was a critical prognostic factor of paediatric neuroblastoma.

### A higher enrichment score of the *TP53* signalling pathway is associated with worse clinical outcomes of paediatric neuroblastoma

**Fig. 2:**
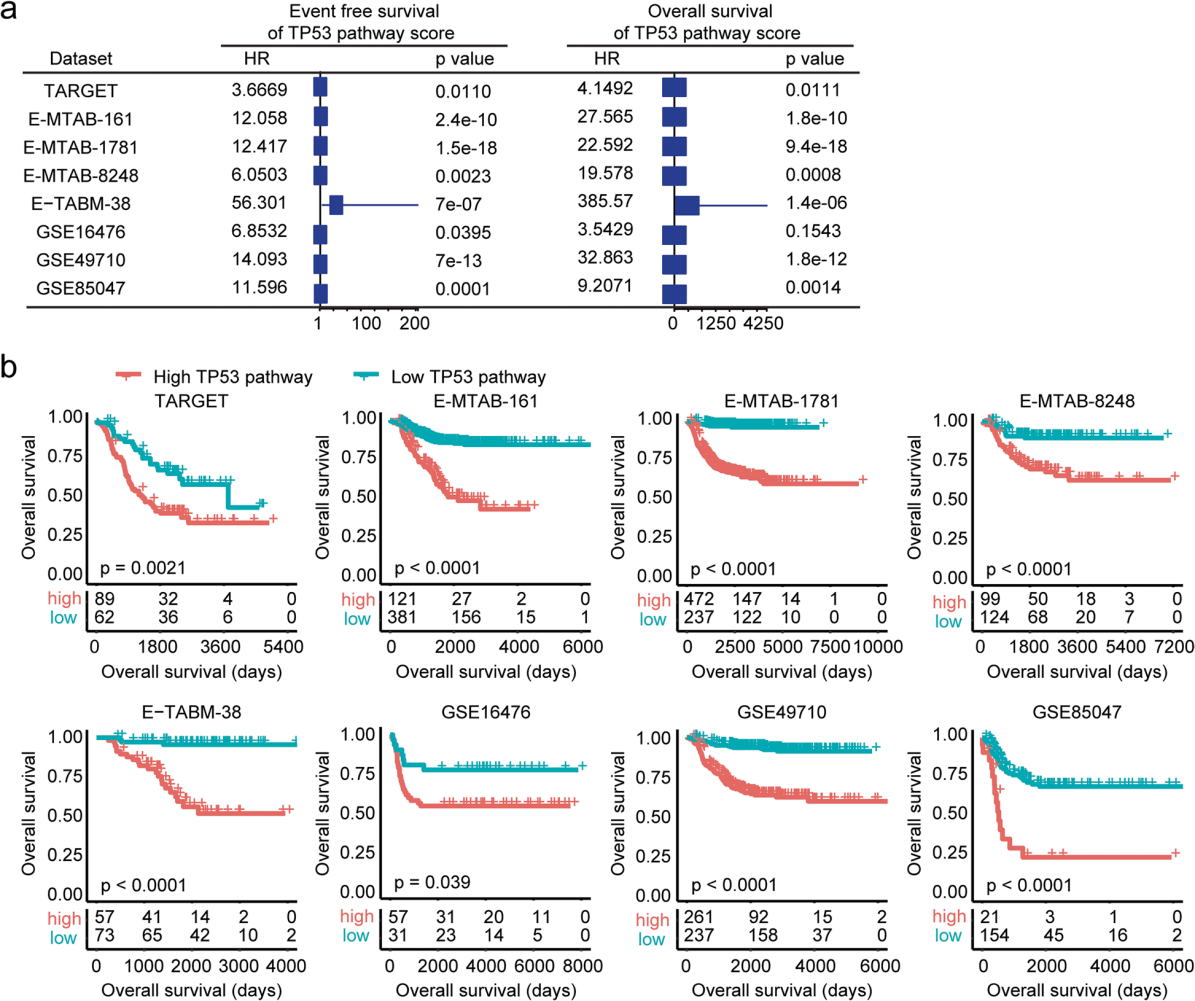
A higher enrichment score of the *TP53* signalling pathway is associated with worse clinical outcomes of paediatric neuroblastoma. **a** Forest plots showing the associations of the *TP53* signalling pathway with neuroblastoma event-free survival and overall survival in the TARGET, E-MTAB-161, E-MTAB-1781, E-MTAB-8248, E-TABM-38, GSE16476, GSE49710 and GSE85047 datasets. **b** Different overall survival of paediatric neuroblastoma patients with higher *TP53* signalling pathway enrichment scores or lower *TP53* signalling pathway enrichment scores in the TARGET, E-MTAB-161, E-MTAB-1781, E-MTAB-8248, E-TABM-38, GSE16476, GSE49710 and GSE85047 datasets

Using the ssGSEA, we determined the enrichment score of the *TP53* signalling pathway in each paediatric neuroblastoma patient. We found that the enrichment score of the *TP53* signalling pathway was associated with the event-free survival and overall survival of paediatric neuroblastoma in the TARGET, E-MTAB-161, E-MTAB-1781, E-MTAB-8248, E-TABM-38, GSE16476, GSE49710 and GSE85047 datasets (Fig. 2a). Moreover, paediatric neuroblastoma patients with lower enrichment scores of the *TP53* signalling pathway had prolonged event-free survival and overall survival in the eight independent datasets (Fig. 2b and Supplementary Fig. 3).

### The *TP53*-associated genes *CCNE1*, *CDK2*, *CHEK2* and *SESN1* are correlated with the clinical outcomes of paediatric neuroblastoma

**Fig. 3:**
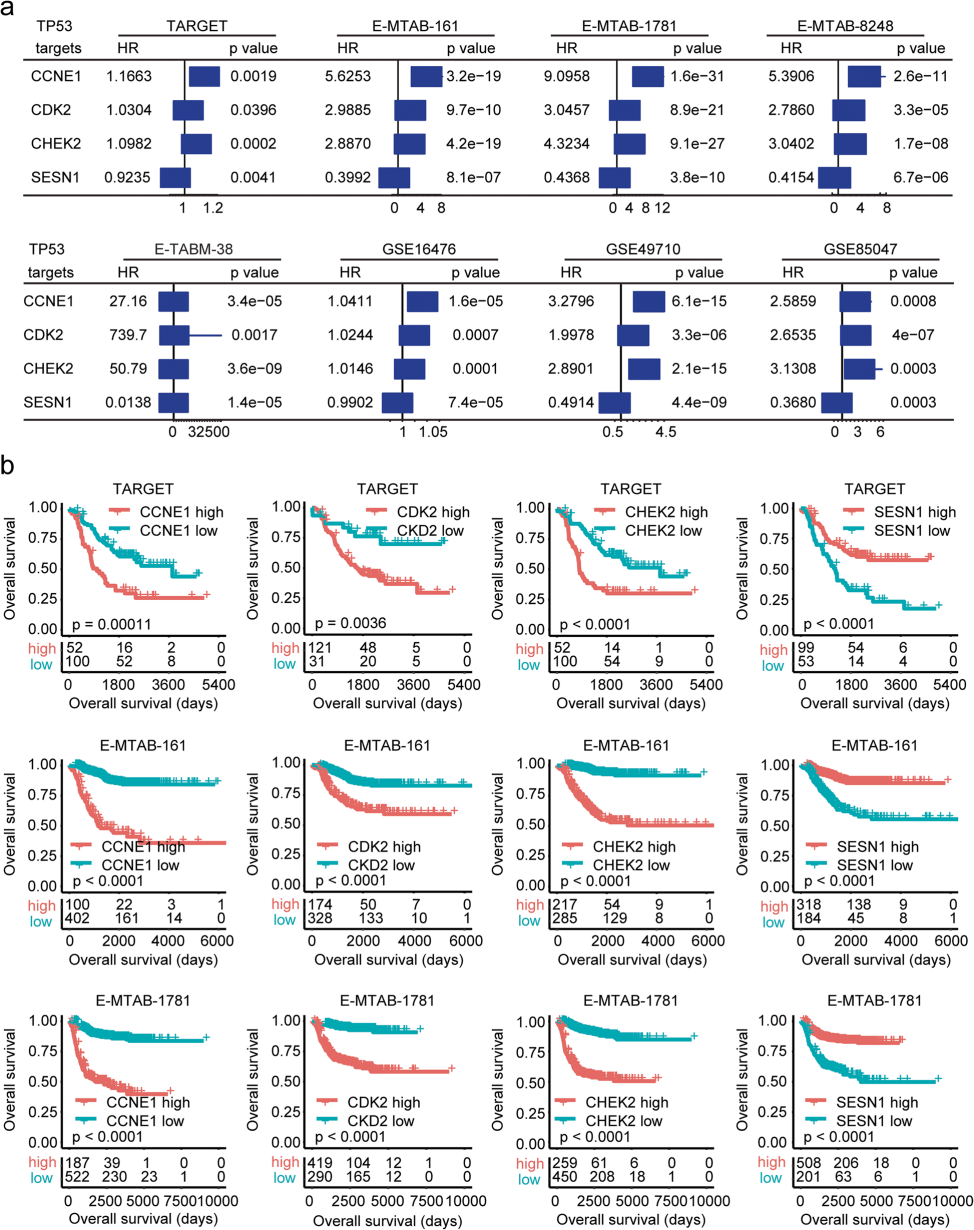
*TP53*-associated genes *CCNE1*, *CDK2*, *CHEK2* and *SESN1* are correlated with the clinical outcomes of paediatric neuroblastoma. **a** Forest plots showing *TP53*-associated genes *CCNE1*, *CDK2*, *CHEK2* and *SESN1* in the prediction of the clinical event-free survival or overall survival of paediatric neuroblastoma in the TARGET, E-MTAB-161, E-MTAB-1781, E-MTAB-8248, E-TABM-38, GSE16476, GSE49710 and GSE85047 datasets. **b** Kaplan–Meier curves showing the prognosis of the *TP53*-associated genes *CCNE1*, *CDK2*, *CHEK2* and *SESN1* in the TARGET, E-MTAB-161 and E-MTAB-1781 datasets

In the GSEA dataset, the *TP53* signalling pathway lists 68 *TP53*-associated genes. We then determined the prognosis of the *TP53*-associated genes in the TARGET, E-MTAB-161, E-MTAB-1781, E-MTAB-8248, E-TABM-38, GSE16476, GSE49710 and GSE85047 datasets. The prognosis of these 68 genes is shown in the supplementary table. Four *TP53*-associated genes, *CCNE1*, *CDK2*, *CHEK2* and *SESN1,* were all detected and were associated with the overall survival of paediatric neuroblastoma in the eight independent datasets (Fig. 3a). However, the prognostic effects of *CCNE1*, *CDK2*, *CHEK2* and *SESN1* were different in paediatric neuroblastoma. Higher expression of *CCNE1*, *CDK2* or *CHEK2* was an unfavourable prognostic factor, while higher expression of *SESN1* was a favourable prognostic factor in paediatric neuroblastoma (Fig. 3a).

The Kaplan–Meier survival analysis further showed that overall survival was decreased in paediatric neuroblastoma patients with high *CCNE1*, *CDK2* or *CHEK2* expression (Fig. 3b and Supplementary Fig. 4). However, overall survival was increased in paediatric neuroblastoma patients with high SESN1 expression in the TARGET, E-MTAB-161, E-MTAB-1781, E-MTAB-8248, E-TABM-38, GSE16476, GSE49710 and GSE85047 datasets (Fig. 3b and Supplementary Fig. 4).

### *TP53*,* CCNE1*, *CDK2*, *CHEK2* and *SESN1* expression is correlated with* MYCN* amplification in neuroblastoma

**Fig. 4:**
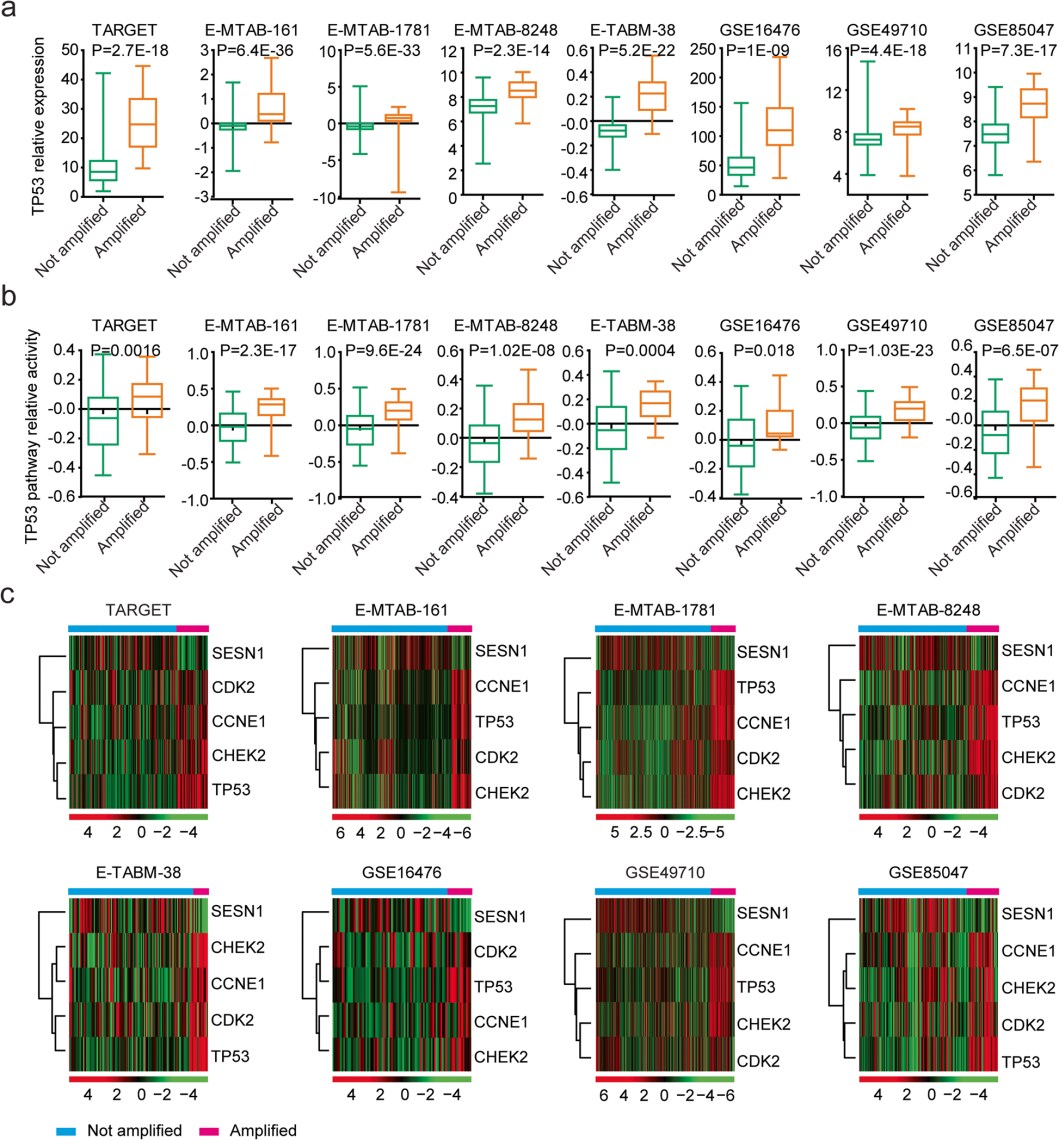
*TP53* and its associated genes are upregulated in *MYCN*-amplified paediatric neuroblastoma patients. **a** Box plots showing the relative *TP53* expression levels in paediatric neuroblastoma patients with or without *MYCN* amplification. **b** Box plots show the relative *TP53* signalling pathway enrichment score in paediatric neuroblastoma patients with or without *MYCN* amplification. **c** Heatmaps demonstrating the different expression levels of *TP53*, *CCNE1*, *CDK2*, *CHEK2* and *SESN1* in paediatric neuroblastoma patients with or without *MYCN* amplification in the TARGET, E-MTAB-161, E-MTAB-1781, E-MTAB-8248, E-TABM-38, GSE16476, GSE49710 and GSE85047 datasets

Previous results showed that *TP53* was a direct target of *MYCN* in paediatric neuroblastoma [21]. Therefore, we determined the associations of *TP53* expression with *MYCN* amplification in paediatric neuroblastoma cohorts. We found that *TP53* mRNA levels were upregulated in *MYCN*-amplified paediatric neuroblastoma patients in the TARGET, E-MTAB-161, E-MTAB-1781, E-MTAB-8248, E-TABM-38, GSE16476, GSE49710 and GSE85047 datasets (Fig. 4a). Moreover, the enrichment score of the *TP53* signalling pathway was also associated with *MYCN* amplification in paediatric neuroblastoma patients (Fig. 4b).

The mRNA expression levels of *CCNE1*, *CDK2*, *CHEK2* and *SESN1* in paediatric neuroblastoma patients with or without *MYCN* amplification were also investigated. Similar to *TP53*, *CCNE1*, *CDK2* and *CHEK2* expression levels were all upregulated in paediatric neuroblastoma patients with *MYCN* amplification (Fig. 4c). In contrast, *SESN1* expression was downregulated in paediatric neuroblastoma patients with *MYCN* amplification in the TARGET, E-MTAB-161, E-MTAB-1781, E-MTAB-8248, E-TABM-38, GSE16476, GSE49710 and GSE85047 independent paediatric neuroblastoma cohorts (Fig. 4c). The results were consistent with overexpression of *CCNE1*, *CDK2* or *CHEK2* being worse prognostic factors, while increased regulation of *SESN1* was a better prognostic factor in paediatric neuroblastoma.

### *CCNE1* and *SESN1* are independent prognostic markers of neuroblastoma

**Fig. 5:**
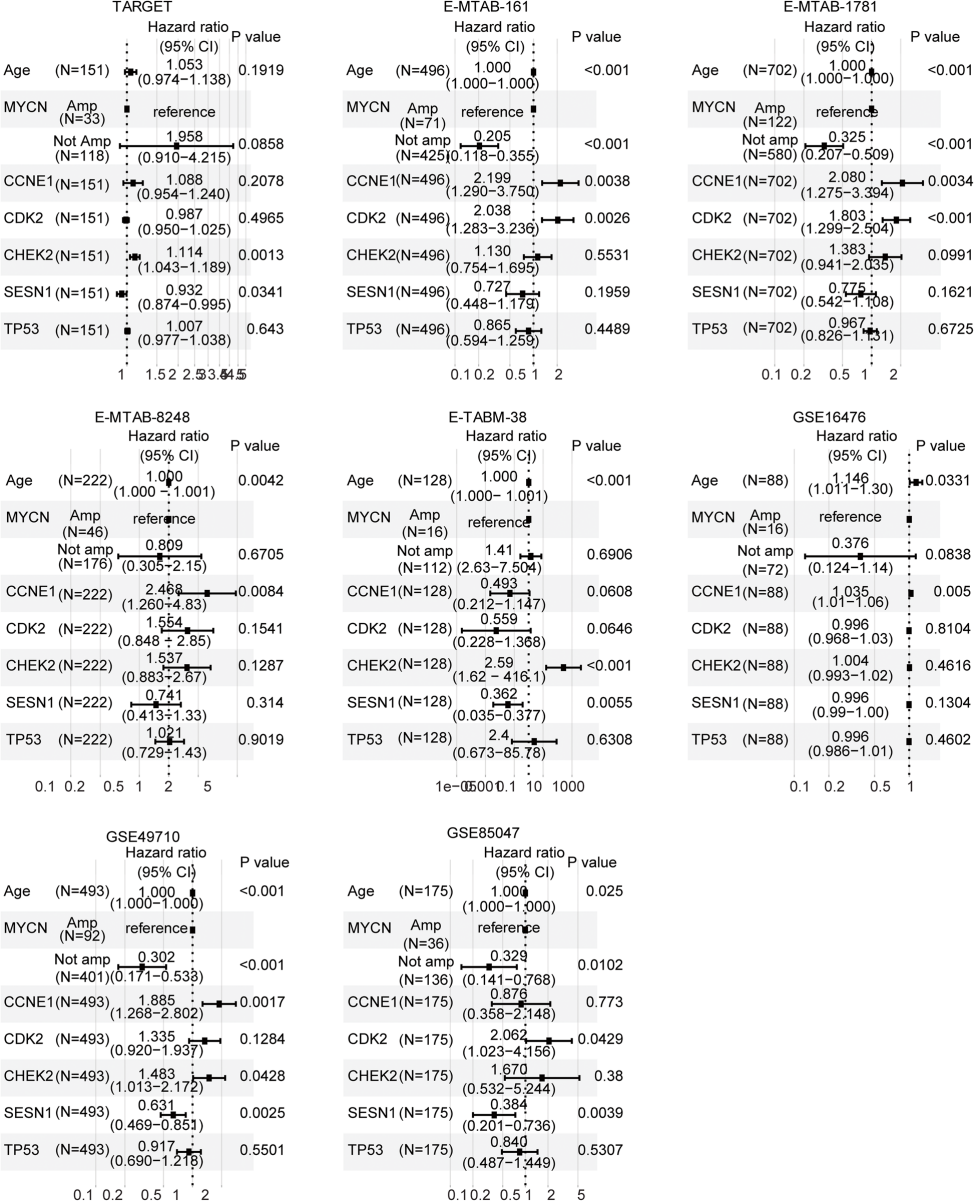
*CCNE1* and *SESN1* are independent prognostic markers of neuroblastoma. Forest plots showing the associations of age, *MYCN* amplification, *TP53*, *CCNE1*, *CDK2*, *CHEK2* and *SESN1* expression with the clinical overall survival of paediatric neuroblastoma in the TARGET, E-MTAB-161, E-MTAB-1781, E-MTAB-8248, E-TABM-38, GSE16476, GSE49710 and GSE85047 datasets

Age at diagnosis and *MYCN* amplification are critical determinants of the clinical outcomes of paediatric neuroblastoma [5]. We then assessed the associations of age at diagnosis, *MYCN* amplification, *TP53*, *CCNE1*, *CDK2*, *CHEK2* and *SESN1* in the prediction of the overall survival of neuroblastoma using a multivariate Cox regression assay. We found that age at diagnosis was an independent prognostic factor of paediatric neuroblastoma in the E-MTAB-161, E-MTAB-1781, E-MTAB-8248, E-TABM-38, GSE16476, GSE49710 and GSE85047 datasets (Fig. 5). *MYCN* amplification was also an independent prognostic factor in the E-MTAB-161, E-MTAB-1781, GSE49710 and GSE85047 datasets (Fig. 5). However, *TP53* was not an independent prognostic factor of paediatric neuroblastoma in any of the eight independent paediatric neuroblastoma cohorts (Fig. 5).

Moreover, *CCNE1* was a prognostic factor of paediatric neuroblastoma in the E-MTAB-161, E-MTAB-1781, E-MTAB-8248, GSE16476 and GSE49710 datasets, independent of *MYCN* amplification and age at diagnosis (Fig. 5). *CDK2* was also an independent prognostic factor of paediatric neuroblastoma in the E-MTAB-161, E-MTAB-1781 and GSE85047 datasets (Fig. 5). *CHEK2* was an independent prognostic factor of paediatric neuroblastoma in the TARGET, E-TABM-38 and GSE49710 datasets (Fig. 5). *SESN1* was an independent prognostic factor of paediatric neuroblastoma in the TARGET, E-TABM-38, GSE49710 and GSE85047 datasets (Fig. 5). Overall, age at diagnosis, *MYCN* amplification, *CCNE1* and *SESN1* were independent prognostic factors in at least four independent paediatric neuroblastoma cohorts.

### Synergistic prognostic effects of *CCNE1* with age at diagnosis in neuroblastoma

**Fig. 6:**
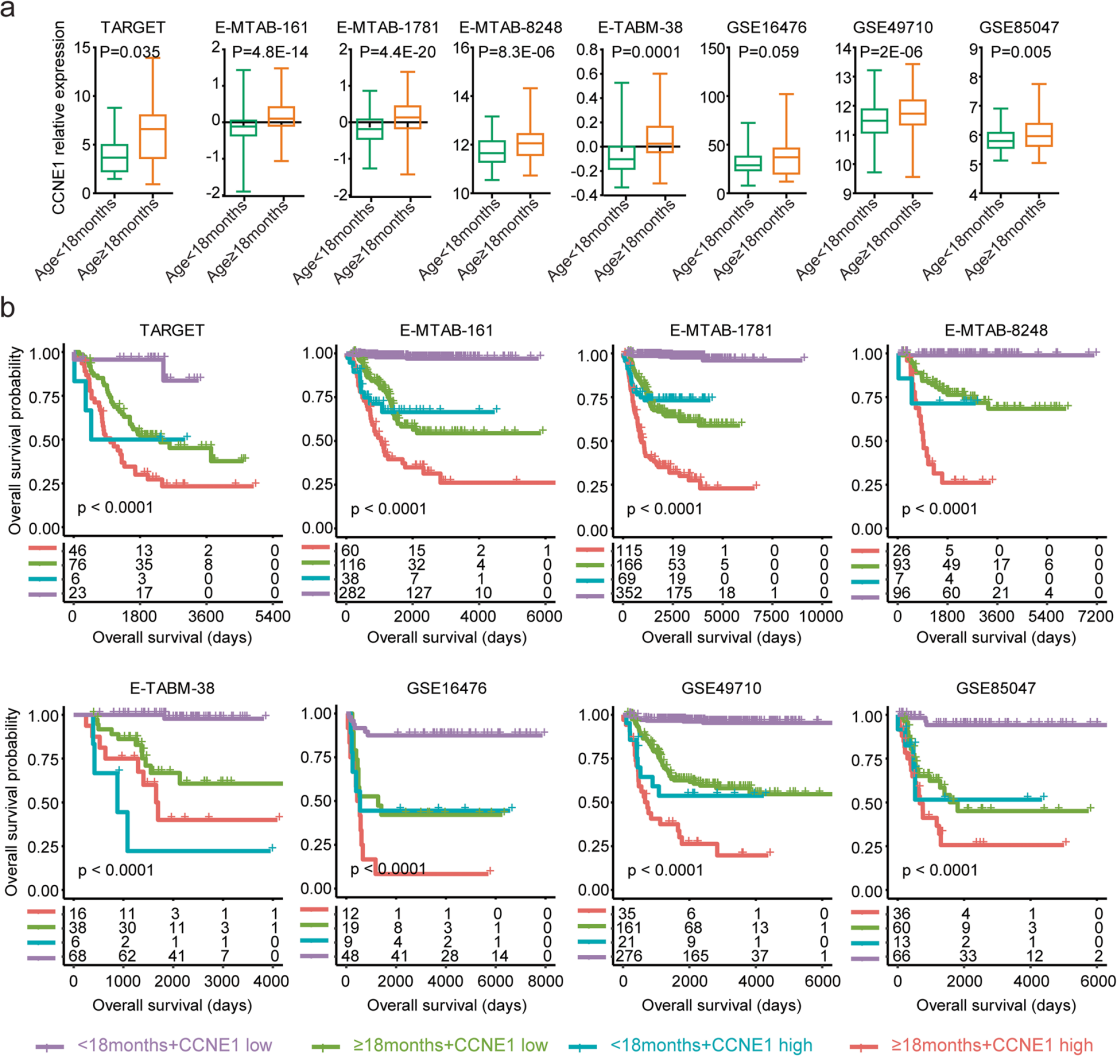
Synergistic prognostic effects of *CCNE1* with age at diagnosis in neuroblastoma. **a** Box plots showing the relative *CCNE1* mRNA expression levels in paediatric neuroblastoma patients with an age at diagnosis ≥ 18 months or < 18 months in the TARGET, E-MTAB-161, E-MTAB-1781, E-MTAB-8248, E-TABM-38, GSE16476, GSE49710 and GSE85047 datasets. **b** Paediatric neuroblastoma patients were divided into four subgroups based on the expression levels of *CCNE1* and age of diagnosis. Different overall survival rates of paediatric neuroblastoma patients in each subgroup were determined

Since *CCNE1* was a prognostic maker of neuroblastoma independent of age at diagnosis, the combinations of *CCNE1* with age at diagnosis could achieve better prognostic effects in patients with paediatric neuroblastoma. First, in the TARGET, E-MTAB-161, E-MTAB-1781, E-MTAB-8248, E-TABM-38, GSE49710 and GSE85047 datasets, the expression levels of *CCNE1* were higher in paediatric neuroblastoma patients with an age at diagnosis ≥ 18 months than in paediatric neuroblastoma patients with an age at diagnosis < 18 months (Fig. 6a).

Furthermore, based on the expression levels of *CCNE1* and age at diagnosis, paediatric neuroblastoma patients in each dataset were divided into four subgroups. Paediatric neuroblastoma patients with an age at diagnosis < 18 months and with *CCNE1* lower expression levels had significantly better prognosis and mostly survived in the following timeframe (Fig. 6b). In contrast, paediatric neuroblastoma patients with an age at diagnosis ≥ 18 months and with higher *CCNE1* expression levels had a significantly worse prognosis (Fig. 6b). Paediatric neuroblastoma patients with an age at diagnosis ≥ 18 months and with *CCNE1* lower expression levels had medium overall survival risks (Fig. 6b). Paediatric neuroblastoma patients with an age at diagnosis < 18 months and with *CCNE1* higher expression levels had the most diverse prognosis than other subgroups (Fig. 6b).

### Synergistic prognostic effects of *SESN1* with age at diagnosis in neuroblastoma

**Fig. 7:**
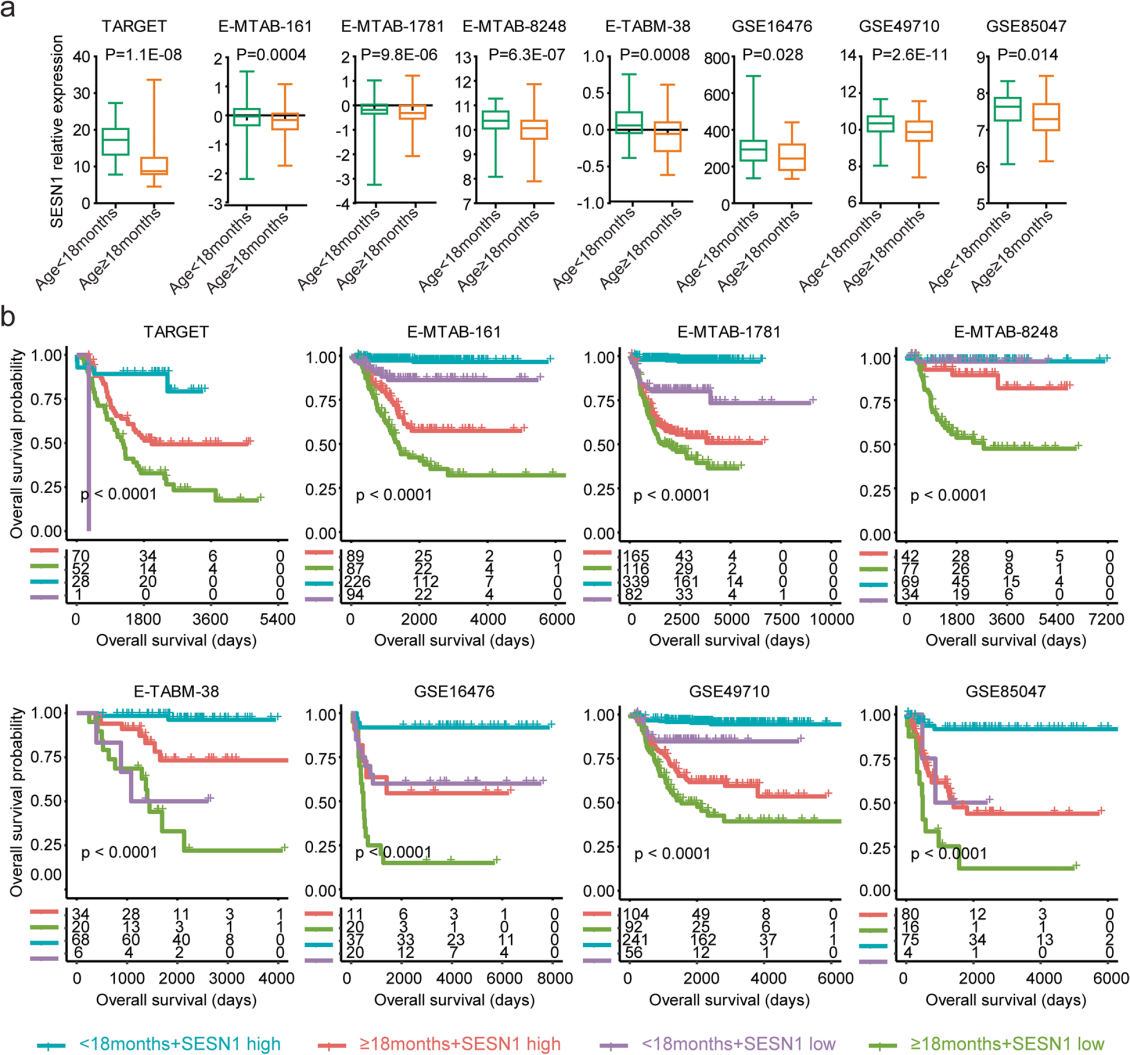
Synergistic prognostic effects of *SESN1* with age at diagnosis in neuroblastoma. **a** Box plots show the relative *SESN1* expression levels in paediatric neuroblastoma patients with an age at diagnosis ≥ 18 months or < 18 months. **b** Paediatric neuroblastoma patients were divided into four subgroups based on the expression levels of *SESN1* and age at diagnosis. Different overall survival rates of paediatric neuroblastoma patients in each subgroup were determined

Consistent with the previous results that overexpression of *SESN1* was a favourable prognostic factor of paediatric neuroblastoma, the expression levels of *SESN1* were lower in paediatric neuroblastoma patients with an age at diagnosis ≥ 18 months in the TARGET, E-MTAB-161, E-MTAB-1781, E-MTAB-8248, E-TABM-38, GSE16476, GSE49710 and GSE85047 datasets (Fig. 7a).

We also observed superior prognostic effects in the combinations of *SESN1* with age at diagnosis in paediatric neuroblastoma. Paediatric neuroblastoma patients with an age at diagnosis < 18 months and *SESN1* higher expression levels had a significantly better prognosis (Fig. 7b). In contrast, paediatric neuroblastoma patients with an age at diagnosis ≥ 18 months and *SESN1* lower expression levels had a significantly worse prognosis (Fig. 7b). Paediatric neuroblastoma patients with an age at diagnosis ≥ 18 months and *SESN1* higher expression levels had medium overall survival risks (Fig. 7b). Paediatric neuroblastoma patients with an age at diagnosis < 18 months and *SESN1* lower expression levels had the most diverse prognosis than other subgroups (Fig. 7b).

### Construction of a nomogram model to predict the overall survival of paediatric neuroblastoma based on age and *TP53*, *CCNE1*, *CDK2*, *CHEK2* and *SESN1* expression

**Fig. 8:**
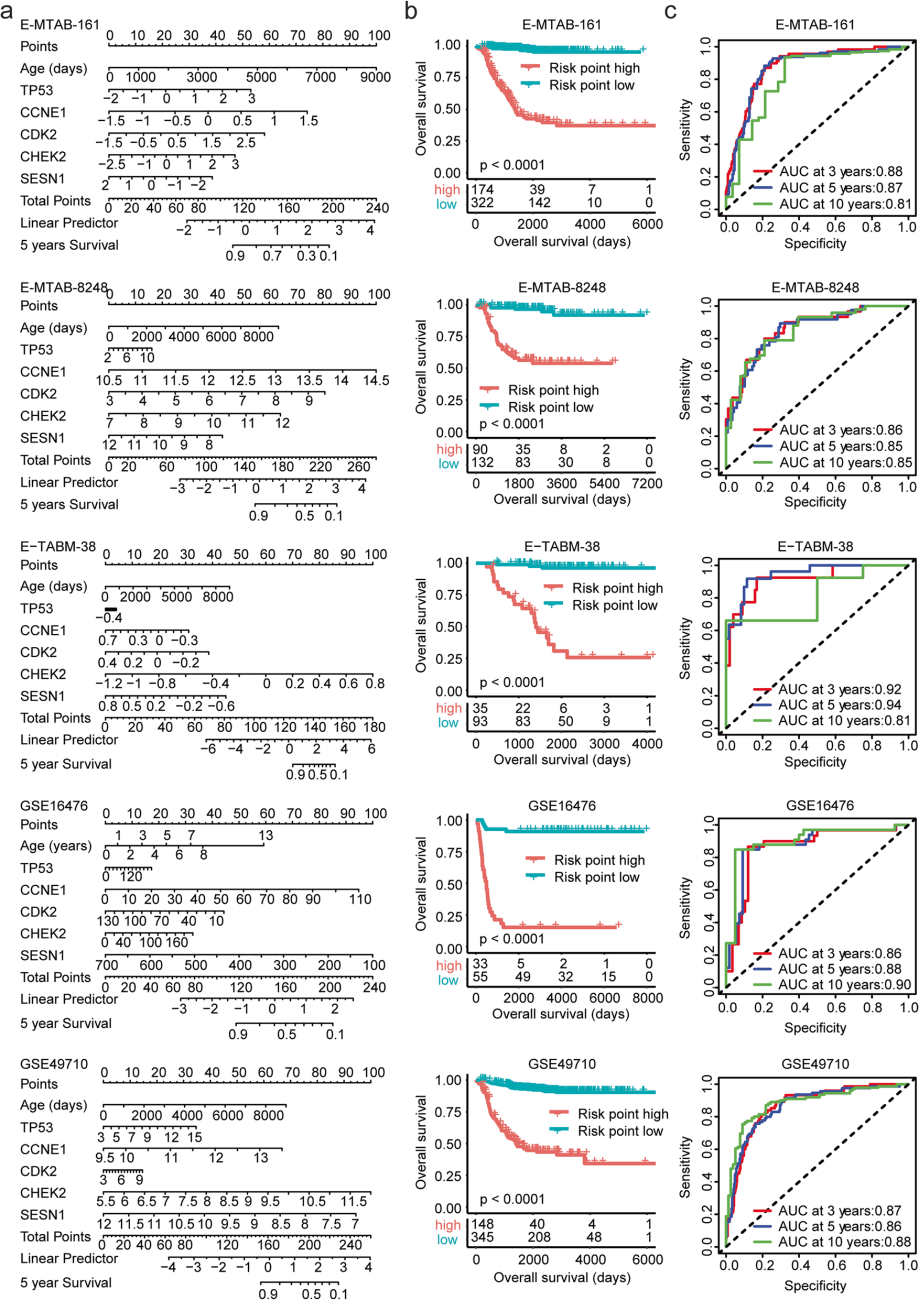
Construction of a nomogram risk model of paediatric neuroblastoma based on age and *TP53 CCNE1*, *CDK2*, *CHEK2* and *SESN1* expression. **a** The nomogram model based on age and *TP53 CCNE1*, *CDK2*, *CHEK2* and *SESN1* expression levels in the E-MTAB-161, E-MTAB-8248, E-TABM-38, GSE16476 and GSE49710 datasets. **b** Kaplan–Meier curves showing the different overall survival rates of paediatric neuroblastoma patients between the low-risk subgroup and the high-risk subgroup. **c** The ROC curves showed the predictive specificity and sensitivity of the nomogram model. The AUC was calculated in the prediction of the three-year, five-year or ten-year overall survival of paediatric neuroblastoma

Our results suggested that *TP53* and its associated genes *CCNE1*, *CDK2*, *CHEK2* and *SESN1* were all associated with the overall survival of paediatric neuroblastoma. We then constructed a nomogram model based on age at diagnosis and *TP53*, *CCNE1*, *CDK2*, *CHEK2* and *SESN1* expression features to predict the clinical overall survival of paediatric neuroblastoma (Fig. 8a). The risk point of each paediatric neuroblastoma patient in the E-MTAB-161, E-MTAB-8248, E-TABM-38, GSE16476 and GSE49710 datasets was obtained in the nomogram model. Paediatric neuroblastoma in the lower risk subgroup had significantly longer overall survival (Fig. 8b). Moreover, the ROC analysis in the E-MTAB-161, E-MTAB-8248, E-TABM-38, GSE16476 and GSE49710 datasets indicated that the nomogram model could predict the three-year, five-year or ten-year overall survival of paediatric neuroblastoma with high specificity and sensitivity (Fig. 8c).

### The nomogram model could predict the overall survival of *MYCN* nonamplified paediatric neuroblastoma

We previously showed that the expression levels of *TP53*, *CCNE1*, *CDK2*, *CHEK2* and SESN1 were correlated with *MYCN* amplification in neuroblastoma patients. We further tested whether the *TP53*-based nomogram model could discriminate paediatric neuroblastoma cases with unfavourable outcomes among *MYCN* nonamplified patients. *MYCN* nonamplified patients from the TARGET, E-MTAB-161, E-MTAB-8248, E-TABM-38, GSE16476 and GSE49710 datasets were selected for investigation. *MYCN* nonamplified paediatric neuroblastoma patients with higher risk points had significantly shortened overall survival (Supplementary Fig. 5). Moreover, the ROC analysis in the TARGET, E-MTAB-161, E-MTAB-8248, E-TABM-38, GSE16476 and GSE49710 datasets showed that the nomogram model could predict the overall survival of *MYCN* nonamplified paediatric neuroblastoma (Supplementary Fig. 5).

## Discussion

Loss of *TP53* function is detected in 50% of adult tumour patients and confers high metastasis and poor clinical outcomes [31]. However, in paediatric neuroblastoma, we showed that overexpression of *TP53* was associated with worse clinical event-free survival and overall survival. Moreover, a higher enrichment score of the *TP53* signalling pathway was also associated with worse clinical outcomes of paediatric neuroblastoma. Furthermore, *TP53*-associated genes *CCNE1*, *CDK2*, *CHEK2* and *SESN1* were all prognostic markers of paediatric neuroblastoma. A nomogram model based on age at diagnosis, *TP53*, *CCNE1*, *CDK2*, *CHEK2* and *SESN1* expression features predicted the clinical overall survival of paediatric neuroblastoma. Our results highlighted the critical functions of *TP53* and its associated genes in the regulation of the progression of paediatric neuroblastoma.

Previous results have shown that *CCNE1* is a target of *MYCN* [32] and an unfavourable prognostic marker of neuroblastoma [33]. *CCNE1* kinase inhibitors are potential drugs for *MYCN*-dependent neuroblastoma [34]. Our results were consistent with these observations that *CCNE1* was upregulated in *MYCN*-amplified paediatric neuroblastoma patients, and *CCNE1* overexpression was associated with worse clinical outcomes of paediatric neuroblastoma. Furthermore, our results showed that *CCNE1* was also overexpressed in paediatric neuroblastoma patients with an age at diagnosis ≥ 18 months. *CCNE1* was an independent prognostic factor, and combinations of *CCNE1* expression with age at diagnosis achieved better prognostic effects in neuroblastoma. *CDK2* was also suggested to be an unfavourable prognostic factor for paediatric neuroblastoma [35]. *CDK2* inhibition suppressed the progression of *MYCN*-amplified neuroblastoma [36], and *CDK2* antagonists represent potential therapeutic drugs in *MYCN*-driven neuroblastoma [37, 38]. However, the prognostic value of *CHEK2* and *SESN1* in paediatric neuroblastoma has never been reported.

As a *TP53* target gene, *SESN1* is associated with the DNA damage response and mTOR signalling pathway [39]. In contrast to *CCNE1*, *CDK2* and *CHEK2*, *SESN1* had opposite expression levels and prognostic effects in paediatric neuroblastoma. *SESN1* was downregulated in *MYCN*-amplified paediatric neuroblastoma patients, and *SESN1* overexpression was associated with better clinical outcomes of paediatric neuroblastoma. Furthermore, our results showed that *SESN1* was a prognostic factor independent of age and *MYCN* amplification. *SESN1* was also overexpressed in paediatric neuroblastoma patients with an age at diagnosis ≥ 18 months, and combinations of *SESN1* expression with age at diagnosis achieved better prognostic effects in neuroblastoma. Our results highlighted the new prognostic roles of *SESN1* in paediatric neuroblastoma.

Paediatric neuroblastoma is extremely heterogeneous. Integrated analysis from different cohorts based on different gene expression technologies may provide more robust results [6]. Integrated analysis of eight independent datasets, TARGET, E-MTAB-161, E-MTAB-1781, E-MTAB-8248, E-TABM-38, GSE16476, GSE49710 and GSE85047, suggested that *TP53* and its associated genes *CCNE1*, *CDK2*, *CHEK2* and *SESN1* were significantly correlated with the clinical outcomes of paediatric neuroblastoma. However, those conclusions were generated from published datasets and lacked validation using additional experiments. Therefore, the functions of *TP53* and its associated genes should be further studied in neuroblastoma cells.

## Conclusions

*TP53*, *CCNE1*, *CDK2*, *CHEK2* and *SESN1* were significantly associated with the clinical event-free survival and overall survival of paediatric neuroblastoma. Combinations of *CCNE1* and *SESN1* with age at diagnosis achieved superior prognosis of neuroblastoma. A nomogram risk model based on age, *TP53*, *CCNE1*, *CDK2*, *CHEK2* and *SESN1* expression predicted the overall survival of paediatric neuroblastoma with high specificity and sensitivity.

## Methods

### Data collection

TARGET paediatric pancancer studies were collected from St Jude Children’s Research Hospital (https://ocg.cancer.gov/) [40]. The E-MTAB-161 [41–43], E-MTAB-1781 [44], E-MTAB-8248 [45] and E-TABM-38 [46–48] datasets were downloaded from EMBL-EBI (https://www.ebi.ac.uk/arrayexpress/). The GSE16476 [49–51], GSE49710 [52] and GSE85047 [53] datasets were collected from the GEO website (www.ncbi.nlm.nih.gov/geo).

### Univariate and multivariable Cox regression

Univariate and multivariable Cox regression analyses were carried out using the “survival” and “survminer” packages in R software. The forest plots were generated using the “forestplot” and “ggforest” packages in R software. The hazard ratio (HR) and *p* values were determined using Cox regression.

### Kaplan–Meier survival analysis

Kaplan–Meier plots were created using the “survival” and “survminer” packages in R software. Paediatric neuroblastoma patients were divided into “high” or “low” subgroups based on the best cut-off points using the “survminer” package. *P* values were determined using the log-rank test.

### Single-sample Gene Set Enrichment Analysis (ssGSEA)

The *TP53* signalling pathway-associated gene set (c2.cp.kegg.v7.2.symbols) was downloaded from the GSEA website (www.broad.mit.edu/gsea/index.html). The enrichment scores of the *TP53* signalling pathway were determined using the “GSVA” package in R software. “GSVA” in the ssGSEA was an unsupervised method evaluating the enrichment score of the *TP53* pathway in each sample based on the expression of 68 *TP53*-associated genes.

### Heatmap presentation

The expression of *TP53* and its datasets *CCNE1*, *CDK2*, *CHEK2* and *SESN1* in neuroblastoma patients with or without *MYCN* amplification was clustered using the “pheatmap” package in R software.

### Nomogram model

The nomogram models were generated using the “rms” and “ggplot2” packages in R software. The risk score was calculated using the “nomogramFormula” package.

### TimeROC curves

The TimeROC curves were generated using the “timeROC” package in R software. The area under the ROC curve (AUC) was calculated by the “survival” package.

### Statistical analysis

The box plots were generated from GraphPad Prism software. Statistical analysis was performed using the two-tailed paired Student’s t test. A *P* value less than 0.05 was chosen to indicate a statistically significant difference.

## Supplementary Information

**Additional file 1: Figure S1.** Working process to analyze the prognosis of *TP53* and its associated genes in paediatric neuroblastoma. **Figure S2.** Kaplan-Meier curves showed the different event free survival of paediatric neuroblastoma patients with *TP53* higher expressions or lower expressions in TARGET, E-MTAB-161, E-MTAB-1781, E-MTAB-8248, E-TABM-38, GSE16476, GSE49710 and GSE85047 datasets. **Figure S3.** Kaplan-Meier curves showed the different event free survival of paediatric neuroblastoma patients with higher *TP53* signaling pathway enrichment score or lower *TP53* signaling pathway enrichment score in TARGET, E-MTAB-161, E-MTAB-1781, E-MTAB-8248, E-TABM-38, GSE16476, GSE49710 and GSE85047 datasets. **Figure S4.** Kaplan-Meier curves showed the prognosis of *TP53* associated genes *CCNE1*, *CDK2*, *CHEK2* and *SESN1* in E-MTAB-8248, E-TABM-38, GSE16476, GSE49710 and GSE85047 datasets. **Figure S5.** The nomogram model could predict the overall survival of *MYCN* non-amplified paediatric neuroblastoma patients. (a) Kaplan-Meier curves showed the different overall survival of *MYCN* nonamplified paediatric neuroblastoma patients between low-risk sub-group and high-risk sub-group in TARGT, E-MTAB-161, E-MTAB-8248, E-TABM-38, GSE16476 and GSE49710 datasets. (c) The ROC curves showed the prediction of the three years, five years or ten years overall survival of MYCN non-amplified paediatric neuroblastoma.

**Additional file 2.**

## Data Availability

The datasets generated and/or analysed during the current study are available from the TARGET (https://ocg.cancer.gov/), EMBL-EBI (https://www.ebi.ac.uk/arrayexpress/) and the GEO websites (www.ncbi.nlm.nih.gov/geo).

## Abbreviations

TARGET: Therapeutically Applicable Research to Generate Effective Treatments
EMBL-EBI: European Bioinformatics Institute
GEO: Gene Expression Omnibus
HR: Hazard ratio
ssGSEA: Single sample Gene Set Enrichment Analysis
AUC: Area under the ROC curve
