# Supplementary material for: TP53 and TP53-associated genes are correlated with the prognosis of paediatric neuroblastoma
Source: BMC Genom Data. 2022 Jun 2;23:41. doi: 10.1186/s12863-022-01059-5 (PMC9164562; doi:10.1186/s12863-022-01059-5)
Supplement: Supplementary file 1 — Additional file 1: Figure S1. Working process to analyze the prognosis of TP53 and its associated genes in paediatric neuroblastoma. Figure S2. Kaplan-Meier curves showed the different event free survival of paediatric neuroblastoma patients with TP53 higher expressions or lower expressions in TARGET, E-MTAB-161, E-MTAB-1781, E-MTAB-8248, E-TABM-38, GSE16476, GSE49710 and GSE85047 datasets. Figure S3. Kaplan-Meier curves showed the different event free survival of paediatric neuroblastoma patients with higher TP53 signaling pathway enrichment score or lower TP53 signaling pathway enrichment score in TARGET, E-MTAB-161, E-MTAB-1781, E-MTAB-8248, E-TABM-38, GSE16476, GSE49710 and GSE85047 datasets. Figure S4. Kaplan-Meier curves showed the prognosis of TP53 associated genes CCNE1, CDK2, CHEK2 and SESN1 in E-MTAB-8248, E-TABM-38, GSE16476, GSE49710 and GSE85047 datasets. Figure S5. The nomogram model could predict the overall survival of MYCN non-amplified paediatric neuroblastoma patients. (a) Kaplan-Meier curves showed the different overall survival of MYCN nonamplified paediatric neuroblastoma patients between low-risk sub-group and high-risk sub-group in TARGT, E-MTAB-161, E-MTAB-8248, E-TABM-38, GSE16476 and GSE49710 datasets. (c) The ROC curves showed the prediction of the three years, five years or ten years overall survival of MYCN non-amplified paediatric neuroblastoma. [file 12863_2022_1059_MOESM1_ESM.docx]

Supplementary figures


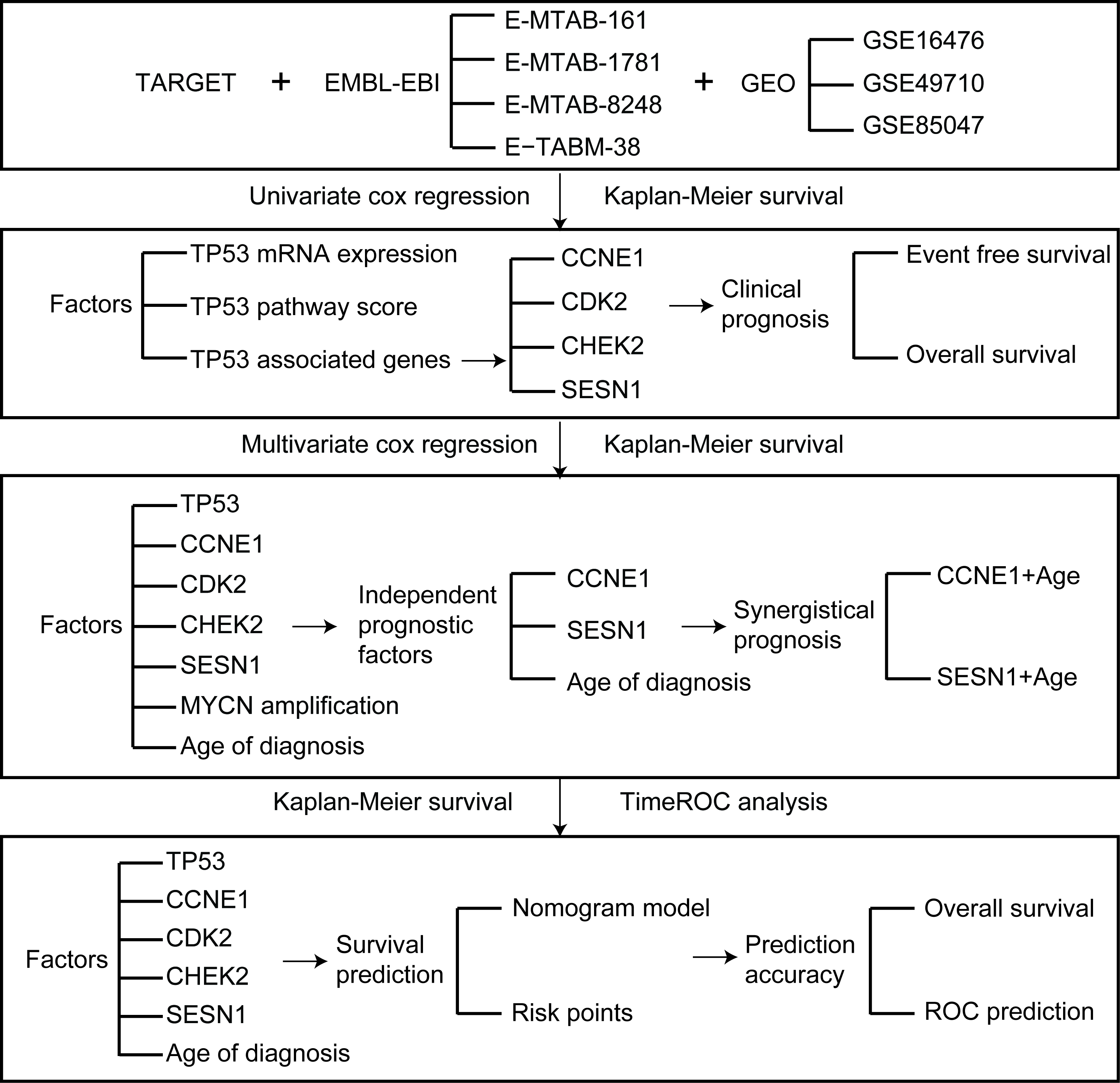


Supplementary Fig. 1 Working process to analyze the prognosis of *TP53* and its associated genes in paediatric neuroblastoma.


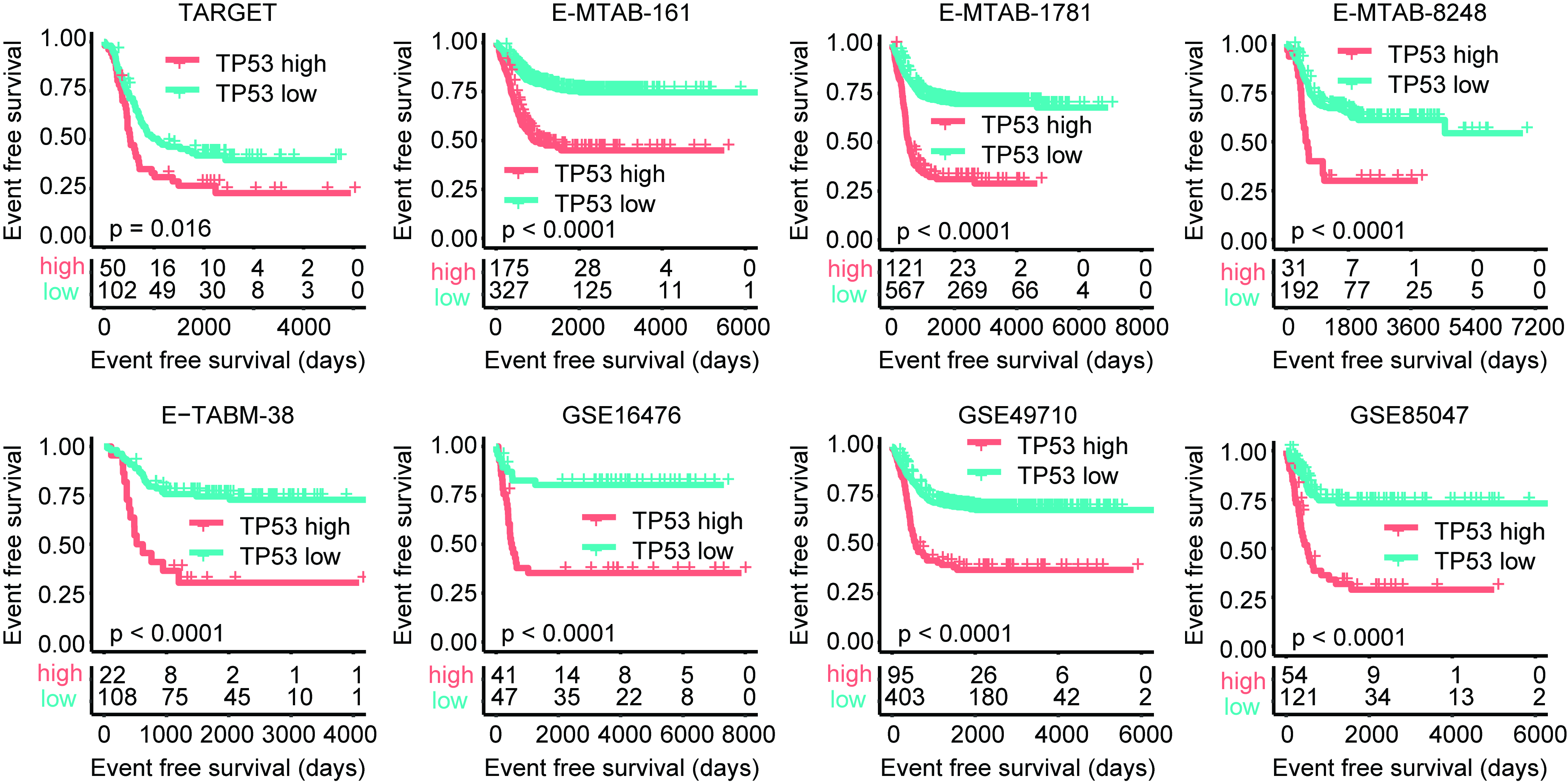


Supplementary Fig. 2 Kaplan-Meier curves showed the different event free survival of paediatric neuroblastoma patients with *TP53* higher expressions or lower expressions in TARGET, E-MTAB-161, E-MTAB-1781, E-MTAB-8248, E-TABM-38, GSE16476, GSE49710 and GSE85047 datasets.


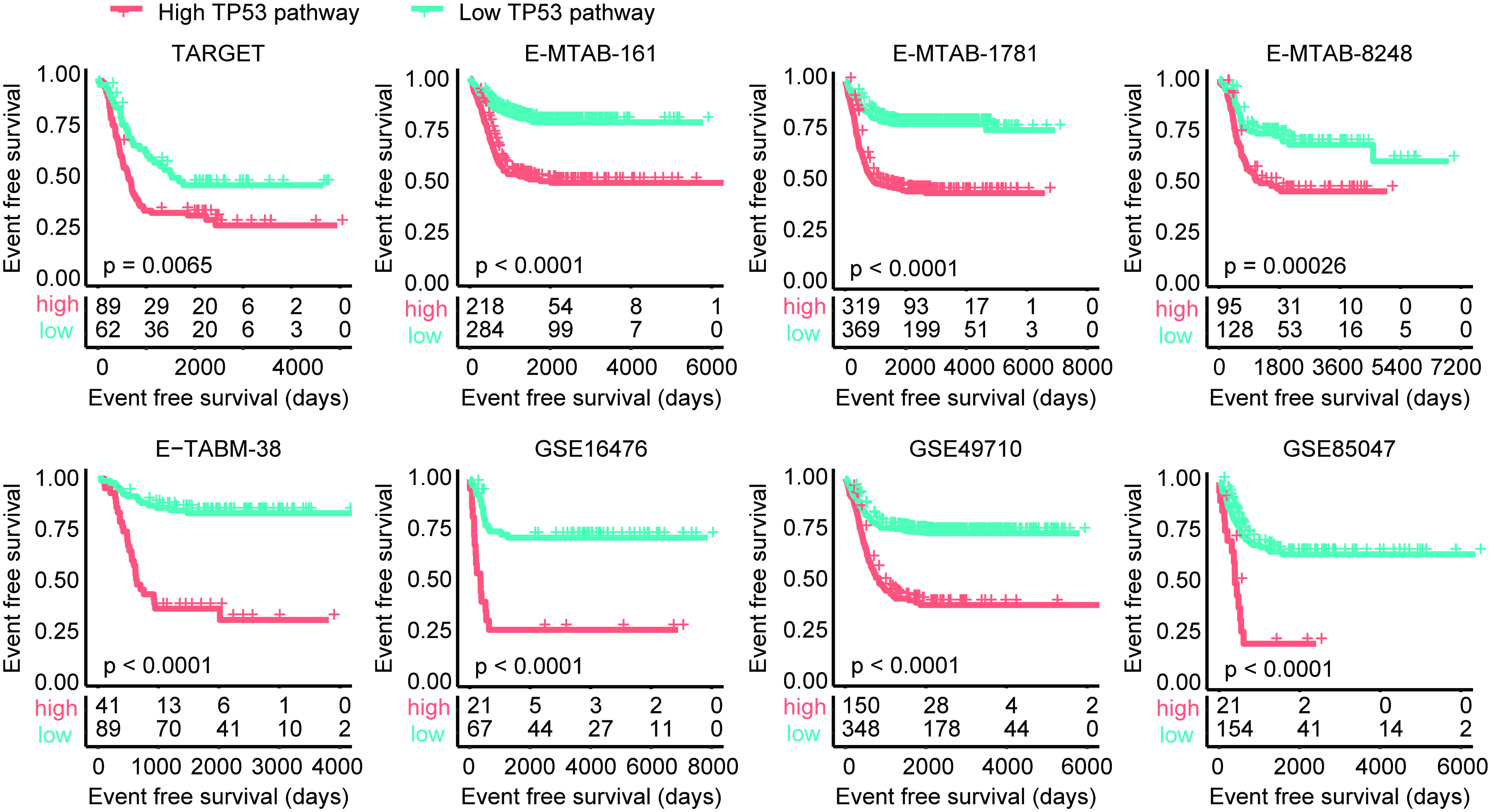


Supplementary Fig. 3 Kaplan-Meier curves showed the different event free survival of paediatric neuroblastoma patients with higher *TP53* signaling pathway enrichment score or lower *TP53* signaling pathway enrichment score in TARGET, E-MTAB-161, E-MTAB-1781, E-MTAB-8248, E-TABM-38, GSE16476, GSE49710 and GSE85047 datasets.


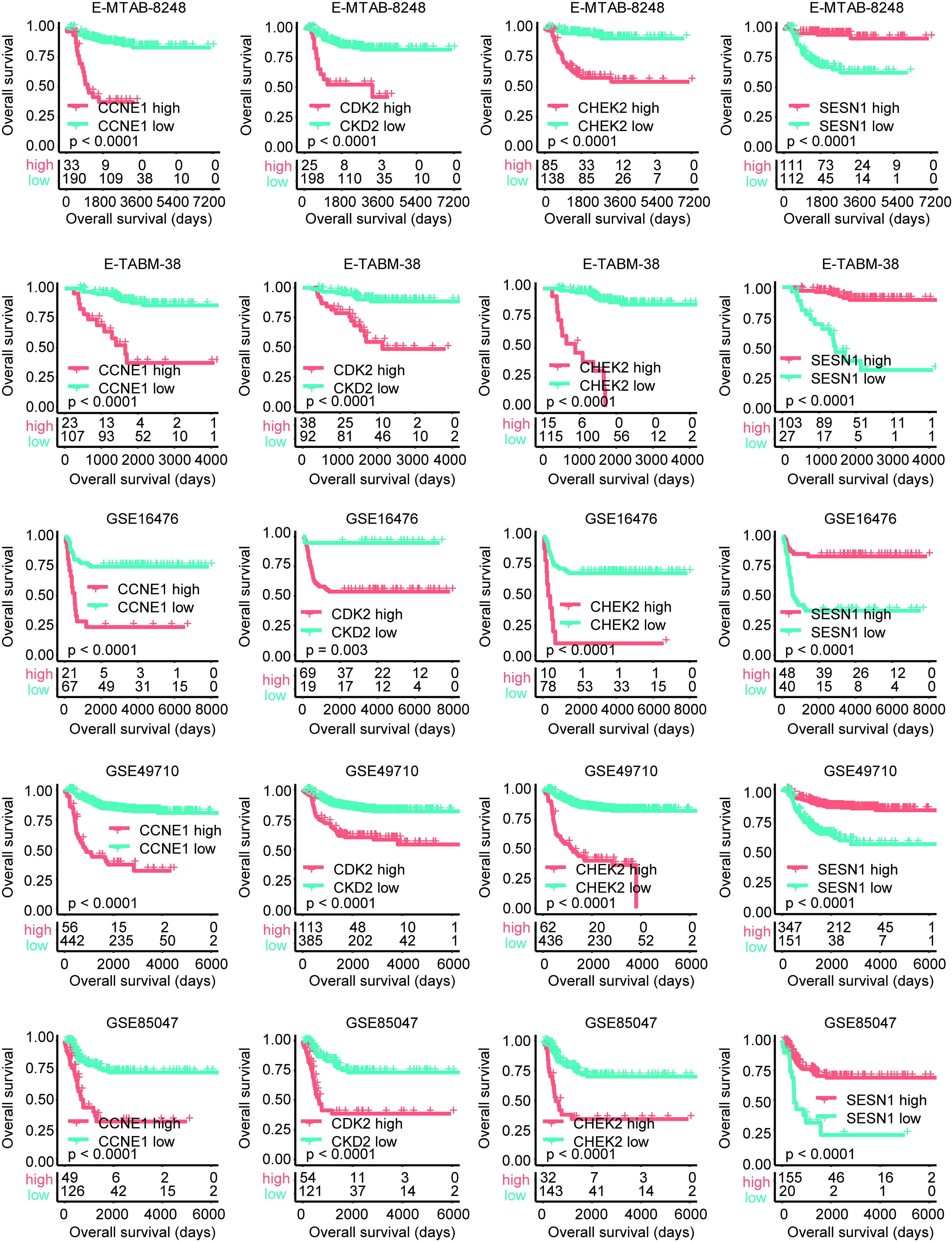


Supplementary Fig. 4 Kaplan-Meier curves showed the prognosis of *TP53* associated genes *CCNE1*, *CDK2*, *CHEK2* and *SESN1* in E-MTAB-8248, E-TABM-38, GSE16476, GSE49710 and GSE85047 datasets.


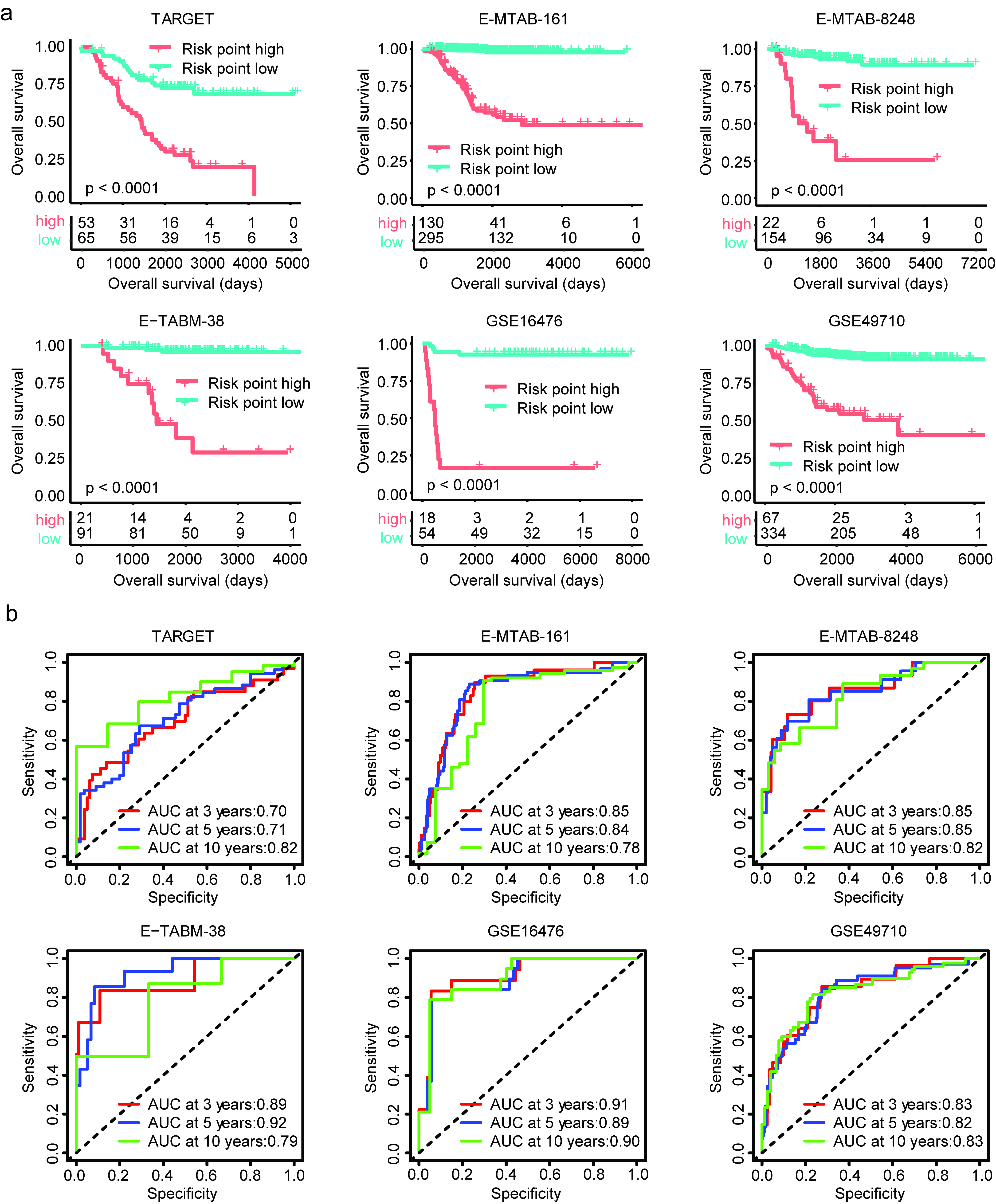


Supplementary Fig. 5 The nomogram model could predict the overall survival of *MYCN* non-amplified paediatric neuroblastoma patients. (a) Kaplan-Meier curves showed the different overall survival of *MYCN* nonamplified paediatric neuroblastoma patients between low-risk sub-group and high-risk sub-group in TARGT, E-MTAB-161, E-MTAB-8248, E-TABM-38, GSE16476 and GSE49710 datasets. (c) The ROC curves showed the prediction of the three years, five years or ten years overall survival of MYCN non-amplified paediatric neuroblastoma.
